# Factors Affecting Hemodialysis Adequacy in Cohort of Iranian Patient with End Stage Renal Disease

**DOI:** 10.5539/gjhs.v8n8p50

**Published:** 2015-12-17

**Authors:** Hosein Shahdadi, Abbas Balouchi, Zahra Sepehri, Hosein Rafiemanesh, Awad Magbri, Fereshteh Keikhaie, Ahmad Shahakzehi, Azizullah Arbabi Sarjou

**Affiliations:** 1 School of Nursing and Midwifery, Zabol University of Medical Sciences, Zabol, IR Iran; 2 Msc Student, Department of Medical-Surgical, Student Research Committee (SRC), School of Nursing and Midwifery, Zabol University of Medical Sciences, Zabol, IR Iran; 3 Department of Internal Medicine, Zabol University of Medical Sciences, Zabol, IR Iran; 4 MSc, School of Public Health, Tehran University of Medical Sciences, Tehran, IR Iran; 5 MD, FACP, FASDIN, Dialysis Access Center of Pittsburgh, PA, USA; 6 Community-Based Nursing Research Center, Zahedan University of Medical Sciences, Zahedan, IR Iran; 7 Student Research Committee, School of Nursing and Midwifery, Iranshahr University of Medical Sciences, Iranshahr, IR Iran; 8 Health Promotion Research Center, Zahedan University of Medical Sciences Zahedan, IR Iran

**Keywords:** dialysis, adequacy, vascular, access, device, hemodialysis, filter

## Abstract

**Background:**

There are many factors that can affect dialysis adequacy; such as the type of vascular access, filter type, device used, and the dose, and rout of erythropoietin stimulation agents (ESA) used. The aim of this study was investigating factors affecting Hemodialysis adequacy in cohort of Iranian patient with end stage renal disease (ESRD).

**Methods:**

This is a cross-sectional study conducted on 133 Hemodialysis patients referred to two dialysis units in Sistan-Baluchistan province in the cities of Zabol and Iranshahr, Iran. We have looked at, (the effects of the type of vascular access, the filter type, the device used, and the dose, route of delivery, and the type of ESA used) on Hemodialysis adequacy. Dialysis adequacy was calculated using kt/v formula, two-part information questionnaire including demographic data which also including access type, filter type, device used for hemodialysis (HD), type of Eprex injection, route of administration, blood groups and hemoglobin response to ESA were utilized. The data was analyzed using the SPSS v16 statistical software. Descriptive statistical methods, Mann-Whitney statistical test, and multiple regressions were used when applicable.

**Results:**

The range of calculated dialysis adequacy is 0.28 to 2.39 (units of adequacy of dialysis). 76.7% of patients are being dialyzed via AVF and 23.3% of patients used central venous catheters (CVC). There was no statistical significant difference between dialysis adequacy, vascular access type, device used for HD (Fresenius and B. Braun), and the filter used for HD (p> 0.05). However, a significant difference was observed between the adequacy of dialysis and Eprex injection and patients’ time of dialysis (p <0.05).

**Conclusion:**

Subcutaneous ESA (Eprex) injection and dialysis shift (being dialyzed in the morning) can have positive impact on dialysis adequacy. Patients should be educated on the facts that the type of device used for HD and the vascular access used has no significant effects on dialysis adequacy.

## 1. Introduction

Chronic Kidney Disease (CKD) is a global problem. Treatment of CKD is costly because of the increase morbidity and mortality in these patients (Astor et al., 2011; Collins et al., 2013; Hoen, Kessler, Hestin, & Mayeux, 1995; Lee et al., 2002). According to the Center for Disease Control and Prevention (CDC), more than 26 million people in America suffer from CKD. They cost 29 million US dollars per annum (Control & Prevention, 2010; Coresh et al., 2007). The prevalence of chronic renal failure in Iran in 2004 was 700,000 people, i.e. 173 cases per 100,000 persons (Nafar et al., 2008). Dialysis treatment causes severe restriction on patient activity, quality of life like weakness, loss of energy, loss of independence, financial stress, sexual disorders, and low self-esteem. It may have also a tremendous impact on the individual and his/her family (Collins et al., 2013). High-adequacy dialysis may ameliorate uremic side effects such as malnutrition, fluid overload, and bleeding and, therefore, mitigate these complications. There has been increased incidence of morbidity in ESRD e.g. deterioration of cognitive functions, depression, peripheral neuropathy, infertility, and increased susceptibility to infection (Adeera Levin, 2006). Dialysis adequacy is measured using Kt/V and URR (urea reduction rate) in which BUN is measured before and after HD (Daugirdas & Schneditz, 1995; Nafar et al., 2008). British renal association (BRA) and Canadian Society of Nephrology (CSN) recommended a KT/V of 1.2 for three times dialysis per week and URR of more than 65% (Kerr & URL, 2005). Various factors affect the adequacy of dialysis including vascular access and the duration of dialysis session (Dhingra, Young, Hulbert-Shearon, Leavey, & Port, 2001). There are three types of vascular access: Arteriovenous fistula (AVF), Arteriovenous graft (AVG) that is made of synthetic and bovine blood vessel, and CVC (Bay, Van Cleef, & Owens, 1998; Hakim & Himmelfarb, 2009). According NKF-DOQI guidelines, the ideal vascular access should have three characteristics: adequate blood flow for dialysis, long life, few side effects (infection, stenosis, thrombosis) (Levin & Rocco, 2006;Sarani et al., 2015; Arbabisarjou & Mahnaz, 2013); AVF meets all these conditions. The CVC, unlike AVF has a high prevalence of infection, high costs, and associated with increased morbidity and mortality (Combe et al., 2000; Hoen et al., 1995; Lee et al., 2002), Therefore, AVF is the recommended vascular conduit for HD by the Canadian and American kidney associations (Levin & Rocco, 2006). CVC is still continued to be used as a bridge of vascular access in most centers (Fadrowski, Hwang, Neu, Fivush, & Furth, 2009; Hayes et al., 2012). Since AVF needs time to mature to be used for HD, CVC are still being used for emergent HD in these patients (Levin & Rocco, 2006). CVC is also used in people with diabetes, old people or females with vascular diseases. It has also been used for patients with heart failure, patients who are waiting for kidney transplant and those who referred to the nephrologists late (Levin & Rocco, 2006; Poredos, Kek, & Verhovec, 1997; Wasse, Speckman, Frankenfield, Rocco, & McClellan, 2007). Health promotion behaviors such as regular exercise, adequate sleep, avoiding alcohol and tobacco use, proper nutrition, avoidance of obesity, medical care and avoidance of stress are advised (Arbabisarjou, Pishkar, & Jahantigh, 2016). The patients like to receive effective and efficient treatments with good outcomes and also, they like to assure that these patients treat and care by clinical specialist as the nurses at the HD wards (Arbabisarjou, 2012). Some studies have shown mixed results for the associations of vascular access with HD adequacy. All these factors along with patients concern about their vascular access prompted us to carry out this study to evaluate the impact of these factors on HD adequacy.

## 2. Methods

### 2.1 Study Design and Participants

This is a cross-sectional study conducted in two dialysis units of government referral hospital in Zabol Cityand Iranshahr city selected from Sistan-Baluchistan province, southeast of Iran. Simple randomization method applied for data collection. A total of 133 (of 150) patients who met the inclusion criteria were included. The inclusion criteria were age over 18 years, those who are on HD for at least 12 months through AVF or CVC, lack of underlying co-morbid conditions that may affect the adequacy of HD. Exclusion criteria were uncooperative patients, patients younger than 18 years, and simultaneous use of two or more vascular accesses for HD, and dialysis intolerance. Patients were monitored from October, 2014 to April 2015. Patients were classified to two groups on the basis of vascular access used for HD (AVF and CVC). Abstraction of data and calculation of Kt/V to measure the adequacy of dialysis were carried out (Levin, 2006). To determine the URR, 2 cc of blood was taken from the arterial line in the first 5 minutes while the pump rate was 50-70 ml/min at the beginning of dialysis. The post HD BUN was analyzed with 2 cc of blood with taken from the venous line in the last five minutes while the pump rate was 50-70 ml/min. Blood amples were sent to the laboratory for analysis. A two-part information questionnaire was used to evaluate factors that might affect HD adequacy. The first part included demographic data: age, gender, marital status, level of education, and the second part included four items: filter type that is classified as high flux and low flux; patient’s shift including morning, afternoon and night; the method of delivery of ESA (Eprex) intravenously through venous line at the end of dialysis or subcutaneous after the end of dialysis; and the device used for dialysis (Fresenius machines made in Germany, B. Braun made in the United States and Nipro made in Germany).

### 2.2 Data Analysis

All data were performed using SPSS for Windows (Version 16.0, SPSS Inc., and Evenston, Illinois). Kolmogorov-Smirnov test was used to test the normality of qualitative data. Descriptive statistics were used to analyze the frequency and frequency percentage and the mean and standard deviation of the demographic characteristics. K-S test, Mann-Whitney and multiple regressions were used when applicable. Statistical significance was considered less than 0.05. Mann-Whitney test was used to compare the adequacy of dialysis in the two groups compared by access type and filter type. The multiple regression tests were used to evaluate the method of delivery of ESA (Eprex) on dialysis adequacy.

### 2.3 Ethics Considerations

The study has been approved by ethics committee of the Zabol University of Medical Sciences. Informed written consent was obtained from each participant in the study before data collection and confidentiality of all responses was maintained through this research. Participation was voluntary and the responses were unidentified.

## 3. Results

A total of 133 Hemodialysis patients were included in the study, 58.60% were male, with age range 18 to 90 years with mean age of 48.24 years (SD 16.50) (Table 1). The range of adequacy of dialysis in the patients under study was 0.28 to 2.39 (units of adequacy of dialysis). The range of dialysis adequacy for the entire study population was 0.91 with a 0.55 interquartile range. The mean of dialysis adequacy in men was 0.90 and in women was 0.92. There was no significant difference between gender and dialysis adequacy (P-Value = 0.81). 76.7% of patients were dialyzed with AVF and 23.3% with CVC. There was no statistical difference in dialysis adequacy between AVF and CVC groups. The filter used has no impact on the dialysis adequacy (Table 2). There was also no difference between the 2 groups in dialysis adequacy as far as device used (P-Value = 0.46) or dialysis shifts (P-Value = 0.67). The route of ESA (EPRIX) injection has reach a close statistical difference for dialysis adequacy (Sc, IV and no type Eprex), (0.87, 0.89 and 1.06), respectively. The difference in HD adequacy between SC route and no Eprex injections had close statistical difference (P-Value = 0.06). When multivariate regression model was applied the relationship between dialysis adequacy and Eprex was statistically significant after controlling for variables such as age, gender, and dialysis machines (P-value = 0.04).

**Table 1:** Distribution of demographic characteristics of patients

Variable (unit)		Frequency	Percentage
Gender	Male	78	58.60
	Female	55	41.40
Marital Status	Single	34	25.6
	Married	99	74.4
Education	Illiterate	74	54.1
	Guidance school	29	21.8
	Diploma	22	16.5
	Associates Degree and BA	10	7.5
Blood Group	A	45	33.8
	B	24	18
	AB	11	8.3
	O	53	39.8

**Table 2:** Investigating the relationship of vascular access type, filter type and device type with dialysis adequacy

Variable (unit)		frequency	percentage	mode of dialysis adequacy (interquartile rang)	P-value
Vascular access	AVF	102	76.7	0.90 (0.60)	0.55
	CVC	31	23.3	0.94 (0.62)	
Filter	High F	59	44.4	0.84 (0.59)	0.34
	Low F	74	55.6	0.94 (0.55)	
Device type	B. Braun	45	34.1	0.91 (0.52)	0.46
	Fresenius	54	40.9	0.95 (0.67)	
	Nipro	33	25	0.80 (0.56)	

## 4. Discussion

This study showed that dialysis adequacy in the groups studied ranges between 0.28 and 2.39 (the unit of adequacy of dialysis) and the mode of dialysis adequacy for the entire study population was 0.91. The values for HD adequacy have been reported as 0.41 ± 1.73 by Debowska et al., 0.27± 1.72 in the study by Ashvandi et al., and 0.31 ± 1.15 in the study by Samakoshi (Samakoosh et al., 2013; Debowska et al., 2014; Oshvandi, Kavyannejad, Borzuo, Gholyaf, & Salavati, 2012). These results were consistent with the current study. The study by Alison et al. are compatible with our study one that there was no difference in dialysis adequacy between AVF and CVC groups who undergo dialysis for at least o year (Ma et al., 2013). In contrast Brazil and Canada (Feldman, Kobrin, & Wasserstein, 1996; Sesso et al., 2007) as well as some other studies showed that people using AVF access have better dialysis adequacy than those with CVC (Allon & Robbin, 2002; Ethier et al., 2008). In patients on chronic Hemodialysis, increasing dialysis sessions, maintaining an ideal vascular access that provides adequate blood flow, and minimizing infection rate are very essential (Ma et al., 2013). The use of AVF for HD meets all these objectives and is considered the ideal conduit for HD (Zaritsky et al., 2008). NKF-DOQI guidelines recommend that at least 50% of dialysis patients should have AVF as the access used for HD (Hoen et al., 1995). In USA 29% of patients use CVC and 40% use AVF method (Levin & Rocco, 2006). But for obvious reasons many patients use CVC as their main access for HD (Fadrowski et al., 2009; Konner, 2003; Lok & Oliver, 2003). One of the main reasons for the inability to use the AVF is high costs for the patient and the healthcare system and referral of patients to nephrologists late or on emergent basis (Mendelssohn, Kua, & Singer, 1995). Also, studies have shown that people who are referred to the nephrologists sooner, have a good chance of having a permanent access placed than those who referred late (Ethier et al., 2008). It seems that due to lack of knowledge and awareness (56%), and residing far from hospitals and dialysis facility (54%) contributes to late referral and therefore, the frequent use of CVC for HD (Chao, Lai, Huang, Chiang, & Huang, 2015). The type of filter used for HD may affect dialysis adequacy. In this study, there was no difference between low flux and high flux filters on dialysis adequacy, which is in line with the study of Garabed (Eknoyan et al., 2002). Other studies have shown different results (Chao et al., 2015; Moslem, Naghavi, & Basiri Moghadam, 2008; Port et al., 2001). When comparing dialysis adequacy among the 3 shifts (morning, afternoon and night), there are no difference. However, other studies have shown that dialysis adequacy is better in the morning followed by night and finally in the afternoon (Fagugli et al., 2006). Patients who have their dialysis during the day (morning and afternoon) have more controlled hypertension, less left ventricular hypertrophy, reduced resistance to erythropoietin treatment and better quality of life than those who undergo dialysis at night (Perl & Chan, 2009). This study showed no difference among the devices used for HD, to our knowledge we could not find any study to confirm or refute our findings (Lambie, Taal, Fluck, & McIntyre, 2004). There was statistically significant difference between the route of ESA delivery and dialysis adequacy, which is consistent with the study conducted by Ebrahimi et al. (Ebrahimi, Khosravi, & Bolbolhaghighi, 2008), but contrary to the study conducted by Zeraati et al. (Zeraati, Naghibi, & Jabari, 2008). Since this study was conducted on a small sample and in two centers close to each other, it is suggested to replicate it on a larger sample and in centers in separate geographical locations.

## 5. Conclusion

Subcutaneous ESA (Eprex) injection and dialysis shift (being dialyzed in the morning) can have positive impact on dialysis adequacy. The device used for HD, and the type of vascular access have little or no impact on the adequacy of HD. Patients should be educated on the facts that the type of device used for HD and the vascular access used has no significant effects on dialysis adequacy.
